# Trichinosis in France

**Published:** 1881-04

**Authors:** 


TRICHINOSIS IN FRANCE.—Dr. Laboulbène, begins thus his relation of the first cases of *trichinosis* observed in France, (read before the *Académie*, Feb. 15). The *Académie* has certainly been impressed by the numerous cases of *trichinosis* observed in the lower animals and in men in later times, in many countries of Europe and in France. Newspapers from Sweden, Spain and America mention the discovery of *trichinæ* in many localities in which they had not been found anteriorly. Follow these quotations :

"Trichinosis, which, till lately, had been chiefly observed in the north of Europe, England and Germany, as well as in America, appeared some time ago in Catalonia, and other provinces in Spain, where it caused a natural alarm."—(*La Independencia Medica*, March, 1878.)

A dispatch from Dingelstad, Dec. 5, 1880, to the *Gazette de l'Allemagne du Nord*, says : "An epidemic of *trichinosis* is in progress here. A great many persons are down with it, and already several have died."

The report of the Health Department of Massachusetts for 1880 contains the following from Mr. Billings, a veterinary surgeon of Boston : "Out of 2,701 hogs examined in the space of five months, 154 contained *trichinæ*, an enormous proportion. These hogs came from different points, mostly from the Western States. Three out of 83 hogs' tongues freshly prepared, contained trichinæ."

In *Lyon Médical*, Jan. 2, 1881, Dr. Cazeneuve mentioned that Mr. Le Clerc, inspector, found trichinæ in slices of pork shipped from New York, which reached Lyon, Nov. 20, 1880. Six per cent. of the slices examined were infected with *trichinæ*.

Mr. Bascou, a veterinary inspector of Paris has found *trichinæ* in American pork.

The first epidemic of *trichinosis* in France, scientifically investigated, was one at Crépy-en-Valois, in 1878. The diseased hog had been reared in France ; 21 persons ate of its flesh ; 17 fell sick, and but one died. A long discussion in the Academy brought out the fact that much more severe epidemics have occurred formerly, which were called *acrodynia*, or other names. For *prophylaxis*, pork should be sliced thin, and heated to 75° C., thus destroying the *trichinæ*.

